# The effect of training volume and intensity on improvements in muscular strength and size in resistance-trained men

**DOI:** 10.14814/phy2.12472

**Published:** 2015-08-13

**Authors:** Gerald T Mangine, Jay R Hoffman, Adam M Gonzalez, Jeremy R Townsend, Adam J Wells, Adam R Jajtner, Kyle S Beyer, Carleigh H Boone, Amelia A Miramonti, Ran Wang, Michael B LaMonica, David H Fukuda, Nicholas A Ratamess, Jeffrey R Stout

**Affiliations:** 1 Institute of Exercise Physiology and Wellness, University of Central FloridaOrlando, Florida; 2 Health & Exercise Science, The College of New JerseyEwing, New Jersey

**Keywords:** Anabolic hormones, hypertrophy, muscle activation, strength

## Abstract

This investigation compared the effect of high-volume (VOL) versus high-intensity (INT) resistance training on stimulating changes in muscle size and strength in resistance-trained men. Following a 2-week preparatory phase, participants were randomly assigned to either a high-volume (VOL; *n *=* *14, 4 × 10–12 repetitions with ∼70% of one repetition maximum [1RM], 1-min rest intervals) or a high-intensity (INT; *n *=* *15, 4 × 3–5 repetitions with ∼90% of 1RM, 3-min rest intervals) training group for 8 weeks. Pre- and posttraining assessments included lean tissue mass via dual energy x-ray absorptiometry, muscle cross-sectional area and thickness of the vastus lateralis (VL), rectus femoris (RF), pectoralis major, and triceps brachii muscles via ultrasound images, and 1RM strength in the back squat and bench press (BP) exercises. Blood samples were collected at baseline, immediately post, 30 min post, and 60 min postexercise at week 3 (WK3) and week 10 (WK10) to assess the serum testosterone, growth hormone (GH), insulin-like growth factor-1 (IGF1), cortisol, and insulin concentrations. Compared to VOL, greater improvements (*P *<* *0.05) in lean arm mass (5.2 ± 2.9% vs. 2.2 ± 5.6%) and 1RM BP (14.8 ± 9.7% vs. 6.9 ± 9.0%) were observed for INT. Compared to INT, area under the curve analysis revealed greater (*P *<* *0.05) GH and cortisol responses for VOL at WK3 and cortisol only at WK10. Compared to WK3, the GH and cortisol responses were attenuated (*P *<* *0.05) for VOL at WK10, while the IGF1 response was reduced (*P *<* *0.05) for INT. It appears that high-intensity resistance training stimulates greater improvements in some measures of strength and hypertrophy in resistance-trained men during a short-term training period.

## Introduction

Resistance training is an effective tool for stimulating muscle hypertrophy and improving strength. By manipulating acute training variables (i.e., exercise selection and order, intensity, volume, and duration, frequency, and rest intervals), differences in mechanical and metabolic stresses can be imposed (Toigo and Boutellier 2006; Ratamess et al. 2009). As the intensity of resistance exercise increases (resulting in increased activation of fast-twitch muscle fibers), a greater emphasis is placed on mechanical stress (Henneman et al. 1965). In contrast, high-volume (i.e., greater number of repetitions concomitant with the use of short rest intervals) programs elicit greater metabolic stress (Ratamess et al. 2009). A minimum intensity threshold is necessary to maximally stimulate muscle activation for those programs targeting metabolic stress (Kraemer and Ratamess 2005; Ratamess et al. 2009). Thus, metabolic stress is targeted by increasing resistance exercise volume and volume load and by reducing rest intervals between sets (Kraemer and Ratamess 2005; Ratamess et al. 2009). The combination of mechanical and metabolic stress has been shown to increase the potential for muscle damage, and it also appears to be a potent stimulus for inducing muscle hypertrophy and strength increases (Clarkson et al. 1992; Toigo and Boutellier 2006). It has been suggested that high volume, moderate-to-high intensity resistance exercise programs utilizing short rest intervals primarily target muscle hypertrophy with secondary strength increases (Baechle et al. 2008; Ratamess et al. 2009). Conversely, high-intensity, low-volume programs utilizing long rest intervals primarily target muscle strength increases with secondary improvements in muscle hypertrophy (Baechle et al. 2008; Ratamess et al. 2009). However, it has been hypothesized that muscle hypertrophy may increase substantially across a larger spectrum of intensity and volume combinations (Schroeder et al. 2013).

The general recommendation of high-volume, moderate-to-high intensity programs utilizing short rest intervals was based on empirical evidence suggesting that this training paradigm is typically used by bodybuilders (Hackett et al. 2013) during hypertrophy phases of periodized training for athletes (Ratamess et al. 2009), and by studies reporting greater increases in muscle hypertrophy as training volume (i.e., number of sets performed) increases (Kraemer 1997; Kraemer et al. 2000; Marx et al. 2001; Goto et al. 2004). Although major health organizations such as the American College of Sports Medicine have recommended a multitude of loading and volume strategies in a periodized manner for advanced hypertrophy training, the moderate-to-high intensity range (6–12 RM) has been regarded as an effective hypertrophy training zone that thought to provide a sufficient balance of mechanical and metabolic stress to the trainee (Ratamess et al. 2009).

A high training volume is associated with an augmented anabolic hormone response to exercise (Kraemer et al. 1990; Crewther et al. 2008; McCaulley et al. 2009) that thought to provide an enhanced stimulus for muscle hypertrophy (McCall et al. 1999; West and Phillips 2012). However, these studies extrapolated the acute endocrine response to a single bout of resistance exercise to changes in skeletal muscle mass over time or simply related change scores. Although exogenously administered anabolic hormones (i.e., testosterone) have been shown to linearly increase lean tissue accruement (Bhasin et al. 2001), no studies have demonstrated a consistent relationship between elevations in the endogenous anabolic hormone response during resistance exercise using multiple joint structural exercises and increases in muscle mass. Furthermore, investigations comparing the acute anabolic hormone response to different resistance exercise protocols (e.g., traditional hypertrophy, strength, or power designs) have been unable to demonstrate that a greater metabolic stress is more advantageous for stimulating augmented testosterone and IGF-1 responses (Kraemer et al. 1990; Schwab et al. 1993; McKay et al. 2008; McCaulley et al. 2009; West et al. 2009, 2010; Hasani-Ranjbar et al. 2012). Interestingly, high-volume training programs have consistently elicited a greater cortisol response to training (Hakkinen and Pakarinen 1993; Crewther et al. 2008; Buresh et al. 2009; McCaulley et al. 2009; West et al. 2010). Although chronically high levels of cortisol are associated with decreases in lean muscle mass (Crowley and Matt 1996; Schakman et al. 2013), the effects of transient elevations in cortisol thought to mediate, in part, tissue remodeling remains poorly understood. Thus, the hypertrophic impact of acute anabolic hormone elevations remains unclear. An alternative perspective suggests that greater mechanical stress associated with high-intensity resistance training may recruit more fast-twitch muscle fibers and provide a greater stimulus for muscle hypertrophy than the metabolic stress associated with high-volume training (Clarkson et al. 1992; Ratamess et al. 2009), but further research is warranted examining the hypertrophic effects of protocols targeting mechanical and metabolic stress, or a combination of both.

A major research limitation is that most studies examined hypertrophic adaptations in previously untrained or moderately trained populations. Program efficacy differentiation is difficult in untrained populations because untrained individuals respond favorably to a multitude of training stimuli. As the “window of adaptation” decreases during long-term resistance training, more scientific recommendations are needed to properly address program design in trained populations targeting strength and hypertrophy increases (Ratamess et al. 2009). However, studies comparing high-intensity to high-volume training programs in resistance-trained individuals are few. These investigations have shown that high-intensity training is more beneficial for strength enhancement, but similar to high-volume training protocols for enhancing muscle hypertrophy (Brandenburg and Docherty 2002; Schoenfeld et al. 2014). However, some methodological limitations (e.g., program design and hypertrophy assessment) raise questions regarding the efficacy of each program type in stimulating strength and hypertrophy increases. In addition, the endocrine response to training remains unclear. Therefore, the purpose of this study was to compare a moderate intensity, high-volume training program utilizing short rest intervals to a high-intensity, lower volume program utilizing a longer rest intervals in resistance-trained individuals. Muscle strength, hypertrophy, and endocrine responses were measured before and following the 8-week training period. It was our hypothesis that the high-intensity program would produce more comprehensive increases in muscle strength and hypertrophy.

## Materials and Methods

### Experimental design

Prior to the onset of the study, all participants were required to complete a 2-week base training program. Assessments of body composition, muscle morphology, strength, and muscle activation were measured pre- and posttraining. Subsequently, participants were randomly assigned to one of two training groups: a high-intensity, low-volume training group (INT; *n* = 15; 24.7 ± 3.4 years; 90.0 ± 15.3 kg; 179.5 ± 5.6 cm) or a high-volume, moderate intensity training group (VOL; *n* = 14; 24.0 ± 2.7 years; 90.1 ± 11.7 kg; 169.9 ± 29.0 cm). Blood samples were collected on day 1 of week 3 (WK3; the first week of training after the base training period) and week 10 (WK10). Each group completed an 8-week resistance training program (four sessions per week) under the direct supervision of certified strength and conditioning specialists (CSCS).

### Participants

Thirty-three physically active, resistance-trained men (24.0 ± 3.0 years; 90 ± 13.8 kg; 174.9 ± 20.7 cm) agreed to participate in this study. Following an explanation of all procedures, risks, and benefits, each participant provided his informed consent to participate in the study. This investigation was approved by the New England Institutional Review Board. All participants were free of any physical limitations (determined by medical history questionnaire and PAR-Q) and had been regularly participating (at the time of recruitment) in resistance training for a minimum of 2 years (5.7 ± 2.2 years).

A major limitation in previous studies is that the participants’ prior training habits were under reported. For example, if participants were following a similar type program to the one used in the study then one would expect fewer improvements. However, if participants were training using a very different type of program compared to the one used in the study then the change in stimulus could lead to improvements and hyperinflate the efficacy of the program. Thus, it is imperative for hypertrophy research to accurately depict prior training of the participants. Prior to the present investigation, all of the participants described their training habits to be different from the present training regimen in terms of exercise order and groupings. Approximately 82% of the participants described their normal repetition range to be either lower (VOL = 77%) or higher (INT = 87%) than what they were assigned in the study, with about 43% typically using a 6–10 RM range and another 21% using an alternating (or pyramid) structure for specific multiple joint structural and assistance exercises. Additionally, 50% of the participants reported using either longer (VOL = 54%) or shorter (INT = 47%) rest periods, while approximately 29% did not track their rest times previously. The remaining participants employed a similar training scheme (i.e., intensity, volume, and rest) to what they were assigned in the study.

### Preparatory phase of training

All participants completed a base resistance training protocol during the 2 weeks prior to the training intervention (see Table1). This phase encompassed a total of six workouts: four workouts (Monday, Tuesday, Thursday, and Friday) during the first week and two workouts (Monday and Tuesday) during the second week. The purpose of the preparatory training program was to instruct proper lifting technique, familiarize participants with all exercises, and ensure the participants initiated the study with a comparable training base. In comparison to the training intervention groups, the exercises (and their order) were identical but the volume (6–8 RM) and rest intervals (1–2 min) differed.

**Table 1:** Resistance training program

Program variable	Preparatory phase	Volume	Intensity
Exercise prescription			
Training intensity	80–85% 1RM	70% 1RM	90% 1RM
Training volume	4 × 6–8 repetitions	4 × 10–12 repetitions	4 × 3–5 repetitions
Rest time	1–2 min	1 min	3 min
Day 1	Day 2	Day 3	Day 4
Specific exercises			
Back squats	Bench press	Barbell squats	Bench press
Deadlifts	Incline bench press	Deadlifts	Incline bench press
Leg press	Dumbbell flys	Barbell lunge	Incline dumbbell flys
Lat pull downs	Seated shoulder press	Seated row	Seated shoulder press
Barbell bent-over rows	Lateral dumbbell raise	Dumbbell pullover	Lateral dumbbell raise
Barbell biceps curls	Triceps extension	Dumbbell biceps curls	Triceps extension

Volume = Sets × Repetitions.

### Anthropometric and morphologic assessments

Anthropometric and morphologic measurements for all participants were conducted approximately 24 h prior to all strength measures and in the following sequence: height, body mass, body composition, and muscle morphology. Height (±0.1 cm) and body mass (±0.1 kg) were determined using a Health-o-Meter Professional scale (Model 500 KL, Pelstar, Alsip, IL) with the participants standing barefoot, with feet together, and in their normal daily attire. Body composition, lean total body mass, and lean mass of the limbs were determined using whole body–dual energy x-ray absorptiometry (DXA) scans (Prodigy™; Lunar Corporation, Madison, WI). Total body estimates of percent fat (%FAT) and nonbone lean body mass (LBM; ±0.1 kg) were determined using company’s recommended procedures and supplied algorithms. Lean arm mass (ARM; ±0.1 kg) was the sum of lean arm mass from both arms, while lean leg mass (LEG; ±0.1 kg) was similarly calculated, following manual demarcation of these regions of interest. Quality assurance was assessed by daily calibrations performed prior to all scans using a calibration block provided by the manufacturer. All DXA measurements were performed by the same certified radiological technician using standardized subject positioning procedures. Intraclass correlation coefficients (ICC_3,1_), standard error of the measurement (SEM), and minimal difference (MD) values for the ARM (ICC_3,1_ = 0.99, SEM_3,1_ = 0.11 kg, MD = 0.23 kg), LEG (ICC_3,1_ = 0.99, SEM_3,1_ = 0.46 kg, MD = 0.91 kg), LBM (ICC_3,1_ = 0.99, SEM_3,1_ = 0.78 kg, MD = 1.53 kg), and %FAT (ICC_3,1_ = 0.99, SEM_3,1_ = 0.73%, MD = 1.44%) were determined on 10 healthy adults (35.9 ± 13.7 years; 96.7 ± 15.0 kg; 168.0 ± 9.7 cm) using the methodology described above.

### Ultrasonography measurements

Noninvasive skeletal muscle ultrasound images were collected from the dominant thigh, arm, and chest locations of all participants. This technique uses sound waves at fixed frequencies to create in vivo, real time images of the limb musculature. Prior to image collection, all anatomical locations of interest were identified using standardized landmarks for the rectus femoris (RF), vastus lateralis (VL), pectoralis major (PM), and triceps brachii (TB) muscles. The landmarks for the RF and VL were identified along the longitudinal distance over the femur at 50% of the length of each muscle, respectively. The length of the RF was defined as the length between the anterior, inferior suprailiac crest and the proximal border of the patella, while the length of the VL encompassed the distance from the lateral condyle of the tibia to the most prominent point of the greater trochanter of the femur. VL measurement required the participant lay on their side. For landmark identification (and ultrasound measurement) of the PM, the participant was required to continue lying supine but with their dominant shoulder abducted and elbow flexed so that the dominant hand was positioned behind their head. Initially, 50% of the distance between the suprasternal notch and the most inferior point of the body of the sternum was identified. Subsequently, the cross-sectional distance from this point to the lateral most border of the muscle (approximately level with the second rib) was used for measurement. Finally, landmark identification of the TB required the participant to straddle the examination table and internally rotate their dominant shoulder, flex the elbow, and rest their dominant hand upon their thigh. The specific landmark for the TB was identified along the longitudinal distance over the humerus at a position 40% of the distance from the lateral epicondyle to the acromion process of the scapula (Ichinose et al. 1998). Subsequently, the participant resumed laying supine on the examination table for a minimum of 15 min to allow fluid shifts to occur before images were collected (Berg et al. 1993). The same investigator performed all landmark measurements for each participant.

A 12-MHz linear probe scanning head (General Electric LOGIQ P5, Wauwatosa, WI) was coated with water soluble transmission gel to optimize spatial resolution and used to collect all ultrasound images. Collection of each image began with the probe being positioned on (and perpendicular to) the surface of the skin to provide acoustic contact without depressing the dermal layer. Subsequently, the extended field of view mode (gain = 50 dB; image depth = 5 cm) was used to capture panoramic images of the muscular regions of interest. For each region, two consecutive images were collected. Each of these images included a horizontal line (∼1 cm), located above the image, which was used for calibration purposes when analyzing the images offline (Chapman et al. 2008). To capture images of the RF, the participant remained in the supine position, with their legs extended but relaxed. A rolled towel was placed beneath the popliteal fossa of the dominant leg, allowing for a 10° bend in the knee as measured by a goniometer, and the dominant foot secured (Bemben 2002). For the VL, the participant was placed on their side with their legs together and the rolled towel between their needs. Once again, the legs were positioned to allow a 10° bend in the knees, as measured by a goniometer (Bemben 2002). Measurement of the PM required the participant to lay supine, in the fashion described above, while TB measurement required the participant to lay prone with their arms extended, resting at their side. For all muscles of interest, muscle cross-sectional area (CSA) was obtained using a cross-sectional sweep in the axial plane at each location across the muscle. In addition to these measures, longitudinal images at 50% of the muscle length were used to determine muscle thickness (MT) in the RF and VL muscles (Cadore et al. 2012). The same investigator positioned each participant and collected all images.

After all images were collected, the ultrasound data were transferred to a personal computer for analysis via Image J (National Institutes of Health, Bethesda, MD, version 1.45s) by the same technician. To measure CSA, the polygon tracking tool in the Image J software was used to isolate as much lean muscle as possible without any surrounding bone or fascia in the RF, VL, TB, and PM (Cadore et al. 2012). Subsequently, Image J calculated the area contained within the traced muscle image and reported this value in centimeter squared (±0.1 cm^2^). The distance (±0.1 cm) between the superficial aponeurosis to the deep aponeurosis was used to determine MT in the RF and VL muscles. The values averaged from both analyzed images (in a specific region) were used for statistical analysis. Prior to the investigation, ICC_3,K_, SEM, and MD values for the RF (MT: ICC_3,K_ = 0.93, SEM_3,K_ = 0.17, MD = 0.45 cm; CSA: ICC_3,K_ = 0.88, SEM_3,K_ = 1.78, MD = 4.60 cm^2^), VL (MT: ICC_3,K_ = 0.88, SEM_3,K_ = 0.16, MD = 0.42 cm; CSA: ICC_3,K_ = 0.99, SEM_3,K_ = 1.11, MD = 3.05 cm^2^), PM (ICC_3,K_ = 0.98, SEM_3,K_ = 2.86, MD = 7.84 cm^2^), and TB (ICC_3,K_ = 0.97, SEM_3,K_ = 1.28, MD = 3.50 cm^2^) were determined on 10 active, resistance-trained men (25.3 ± 2.0 years; 90.8 ± 6.8 kg; 180.3 ± 7.1 cm) using the methodology described above.

### Strength testing

Strength in the bench press and squat exercises was assessed pre- and posttraining. Participants were scheduled for testing at a standard time of day similar to their training schedule. A general warm up consisting of riding a cycle ergometer for 5 min at a self-selected resistance preceded strength testing. The general warm up was followed by a specific warm up consisting of 10 bodyweight squats, 10 alternating lunges, 10 walking “knee hugs,” and 10 walking “butt kicks.” Standardized procedures were used for the one repetition maximum (1RM) barbell bench press and barbell back squat, respectively (Hoffman 2006). For each exercise, a warm-up set of 5–10 repetitions was performed using 40–60% of the participant’s perceived 1RM. After a 1-min rest period, a set of 2–3 repetitions was performed at 60–80% of the participant’s perceived 1RM. Subsequently, 3–5 maximal trials (one repetition sets) were performed to determine the 1RM. For the bench press, proper technique was enforced by requiring all participants to maintain contact between their feet and the floor; their buttocks, shoulders, and head with the bench; and use a standard grip (slightly wider than shoulder width) on the bar. Upon lowering the bar to their chest, participants were required to pause briefly and wait for an “UP!” signal before initiating concentric movement. The purpose for this pause was to eliminate the influence of bouncing and distinguish eccentric from concentric muscle activation during electromyography analysis. Any trials that involved excessive arching of the back or bouncing of the weight were discarded. For the back squat, a successful attempt required the participant to descend to the “parallel” position, where the greater trochanter of the femur was aligned with the knee. At this point, an investigator located lateral to the participant, provided an “UP!” signal, indicating that proper range of motion had been achieved; no pause was required for the squat exercise. Rest periods between attempts were 2–3 min in length. Upon determining their 1RM for each exercises, each participant was allotted a 5-min rest period before completing three additional one repetition sets with 40%, 60%, and 80% of their 1RM; 1-min rest periods were provided between these sets. All testing was completed under the supervision of a CSCS.

### Electromyography measurements

To assess changes in muscle activation efficiency, electromyography (EMG) data were collected using previously described configurations for bipolar (4.6 cm center-to-center) surface electrode (Quinton Quick-Prep silver-silver chloride) placement (Hermens et al. 1999). Briefly, for the back squat electrodes were placed over the VL (60% of distance between the head of the greater trochanter and lateral aspect of the patella) and RF (50% of distance between the inguinal crease and superior border of the patella) muscles, with the reference electrode placed over the lateral epicondyle of femur. For all participants, electrodes were placed on the dominant side, 2 cm apart, and parallel to the active fibers. The same investigator was used to identify landmarks and place electrodes at PRE and POST. Additionally, the interelectrode impedance was kept below 5000 ohms by shaving, abrading, and cleaning (with alcohol) the placement site prior to testing. EMG signals were obtained using a differential amplifier (MP150 BIOPAC Systems, Inc., Santa Barbara, CA) sampled at 1000 Hz and then transferred to a personal computer for analysis. EMG signals were band-pass filtered from 10 to 500 Hz, rectified (full-wave), and expressed as integrated EMG (iEMG) values (±0.01 V ˙ sec) by software (AcqKnowledge v4.2, BIOPAC Systems, Inc.).

During the back squat, muscle activation data were collected during each participant’s 1RM and subsequent single repetitions at 40%, 60%, and 80% 1RM. The linear regression slope of muscle activation and increasing back squat load was used to describe muscle activation efficiency. Thus, improvements in muscle activation efficiency were determined by comparing the changes in the activation regression slope following the 8-week training program using the same loads assessed at PRE (deVries 1968). In this way, an increase in the slope would indicate greater muscle activation; a common neuromuscular improvement in novice lifters. However, a decrease (or reduction) in muscle activation would imply less muscle is necessary for the given task; or greater muscle activation efficiency. Since the absolute loads remained consistent between PRE and POST, an improvement in muscle activation efficiency would be the consequence of muscle hypertrophy occurring in an advanced lifter.

### Resistance training intervention

Participants reported to the Human Performance Lab (HPL) four times per week to complete their assigned training program (see Table1). Briefly, the INT training program required the participants to perform four sets at 3–5 repetitions with 90% of their 1RM, with 3-min rest periods between sets, while the VOL group performed four sets at 10–12 repetitions with 70% of their 1RM, with a 1-min rest period between sets. Both groups performed the same exercises. Training intensity was determined from 1RM testing (bench press and squat) and estimated 1RM (all other exercises) (Brzycki 1993) from each participant’s performance during the preparatory training phase. Progressive overload was achieved by increasing the load when all prescribed repetitions (for a particular exercise) were achieved on two consecutive workouts. Weekly training volume load was calculated as the average of the number of repetitions × load in the squat and bench press exercises. Following each training session, participants were provided ∼235 mL of chocolate milk (170 calories; 2.5 g fat; 29 g carbohydrate; 9 g protein) or Lactaid® (150 calories; 2.5 g fat; 24 g carbohydrate; 8 g protein) for lactose-intolerant participants. All training sessions occurred under the supervision of a CSCS.

### Blood sampling

Blood samples were obtained at four time points: baseline (BL), immediately postexercise (IP), 30 min postexercise (30P), and 60 min postexercise (60P). Participants reported to the HPL 3 h postprandial, at a standardized time of day consistent with their normal training schedule. All blood samples at POST were taken at the same time of day as PRE to avoid the confounding influence of diurnal variations. All blood samples were obtained using a Teflon cannula placed in a superficial forearm vein using a three-way stopcock with a male luer lock adapter and plastic syringe. The cannula was maintained patent using an isotonic saline solution (Becton Dickinson, Franklin Lakes, NJ). Blood samples at BL were obtained following a 15-min equilibration period. Following the BL blood sample, participants were provided ∼235 mL of chocolate milk (170 calories; 2.5 g fat; 29 g carbohydrate; 9 g protein) or Lactaid® (150 calories; 2.5 g fat; 24 g carbohydrate; 8 g protein). Following the resistance exercise protocol, participants remained in the HPL for all subsequent blood draws. IP blood samples were taken within 1 min of exercise cessation. Following IP blood samples, participants were provided their normal ∼235 mL of chocolate milk. Participants were instructed to lie in a supine position for 15 min prior to the 30P and 60P blood draws.

All blood samples were collected into two Vacutainer® tubes, one containing no anticlotting agent (6 mL) and the second containing K_2_EDTA (6 mL). A small aliquot of whole blood was removed and used for determination of hematocrit and hemoglobin concentrations. The blood in the first tube was allowed to clot at room temperature for 30 min and subsequently centrifuged at 3000*g* for 15 min along with the remaining whole blood from the second tube. The resulting plasma and serum were placed into separate microcentrifuge tubes and frozen at −80°C for later analysis.

### Biochemical analysis

Hematocrit concentrations were analyzed from whole blood via microcentrifugation (CritSpin, Westwood, MA) and microcapillary techniques. Hemoglobin concentrations were analyzed from whole blood using an automated analyzer (HemoCue, Cypress, CA). Blood lactate concentrations were analyzed from plasma using an automated analyzer (Analox GM7 enzymatic metabolite analyzer, Analox instruments USA, Lunenburg, MA). Coefficient of variation for each assay was 1.53% for hematocrit, 0.55% for hemoglobin, and 0.98% for blood lactate. Plasma volume shifts were calculated using the formula established by Dill and Costill (1974). To eliminate interassay variance, all samples were analyzed in duplicate by a single technician.

Circulating concentrations of testosterone (TEST), cortisol (CORT), insulin-like growth factor (IGF-1), 22-kD growth hormone (GH), and insulin (INSL) were assessed via enzyme-linked immunosorbent assay (ELISA) and a spectrophotometer (BioTek Eon, Winooski, VT) using commercially available kits. To eliminate interassay variance, all samples for each assay were thawed once and analyzed in duplicate in the same assay run by a single technician. Samples were analyzed in duplicate, with an average coefficient of variation of 3.74% for TEST, 4.03% for CORT, 6.77% IGF-1, 3.50% for GH, and 6.54% for INSL. The area under the curve (AUC), expressed in arbitrary units (au) via the trapezoidal method, was calculated and used to analyze the total training response. Blood variables were not corrected for plasma volume shifts due to the importance of molar exposure at the tissue receptor level.

### Nutrient intake and dietary analysis

Participants were instructed to maintain their normal kilocaloric intake habits throughout the course of the investigation. Kilocaloric and macronutrient intake were monitored via weekly food diaries. Consequently, all participants were required to record all food and beverage intake over the course of 3 days (two weekdays and one weekend day) during the week of PRE (week 2) and POST (week 10). The FoodWorks Dietary Analysis software version 13 (The Nutrition Company, Long Valley, NJ) was used to analyze dietary recalls. For statistical analysis, total caloric, macronutrient (protein, carbohydrate, and fat), and branched chain amino acid (leucine, isoleucine, and valine) intake were analyzed relative to body mass.

### Statistical analysis

To identify differences between training protocols on changes in muscle size, strength, and activation, an analysis of covariance (ANCOVA) was performed on all measures collected at POST. Associated values collected at PRE were used as the covariate to eliminate the possible influence of initial score variances on training outcomes. Following any significant *F* ratio, a paired sample *t*-test was used to determine whether significant difference existed between measures collected prior to and immediately following 8 weeks of training. To examine if the changes could be considered real, all individual changes in body composition measures were compared to their calculated MD (Weir 2005). Using (eq. 1), MD is determined by creating a 95% confidence interval about the standard error of the measurement (SEM). Thus, any change occurring within this confidence interval would be interpreted as being consistent with the measurement error of the test, while changes occurring outside of the interval reflect real changes in body composition.

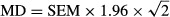
1

To examine group differences in the acute endocrine response to exercise during WK3 and WK10, a repeated measures ANCOVA was performed, where hormone concentrations at baseline (of WK3 and WK10, respectively) were used as the covariate. In the event of a significant interaction, a repeated measures analysis of variance (ANOVA) was performed on each group individually at WK3 and WK10. In the event of a significant *F* ratio, least squared distance (LSD) post hoc analysis was used to determine significant differences between each time point (i.e., IP, 30P, and 60P) and baseline hormone concentrations. The effect of training on AUC measures was also analyzed by a repeated measure ANOVA. In the event of a significant *F* ratio, an independent *t*-test was used to assess group differences at WK3 and WK10.

All between group differences were further analyzed using effect sizes (*η*^2^: Partial eta squared). Interpretations of effect size were evaluated (Cohen 1988) at the following levels: small effect (0.01–0.058), medium effect (0.059–0.137), and large effect (>0.138). A criterion alpha level of *P* ≤ 0.05 was used to determine statistical significance. All data are reported as mean ± standard deviation. Statistical Software (V. 21.0, SPSS Inc., Chicago, IL) was used for all analyses.

## Results

### Resistance training program comparisons

No group differences in absolute strength (1RM; squat: *P* = 0.653; bench press: *P* = 0.661) or relative strength (1RM ˙ body mass^−1^; squat: *P* = 0.308; bench press: *P* = 0.843) were observed prior to the training intervention. Participants completed at least 90% of their respective resistance training sessions over the course of the 8-week training study, and no differences (*P* = 0.547) in the number of workouts completed were observed between groups. The average training volume load was significantly higher (*P* < 0.001) for VOL (squat: 8753 ± 1033 kg; bench press: 4412 ± 729 kg) compared to INT (squat: 4528 ± 889 kg; bench press: 2757 ± 696 kg). The average time to completion for each training session for VOL (68.2 ± 5.6 min) was significantly (*P* < 0.001) faster than INT (95.0 ± 8.7 min).

### Anthropometric and morphological changes

Changes in muscle size and anthropometrics following the training intervention are presented in Table2. Lean arm mass was significantly (*F* = 4.816, *P* = 0.037, *η*^2^ = 0.156) greater in INT (5.2 ± 2.9%, *P* < 0.001) compared to VOL (2.2 ± 5.6%, *P* = 0.314) posttraining. Furthermore, 93.3% of participants in INT experienced a change in lean arm mass that was greater than the minimal difference (MD = 0.23 kg) for this measure. In contrast, only 64.3% of participants in VOL experienced such a change. Although no other significant group differences were observed, a larger percentage of participants (60.0%) in INT compared to VOL (35.7%) experienced changes that exceeded the minimal difference for LBM (MD = 1.53 kg). Similar responses were also noted in lean leg mass (MD = 0.91 kg; INT: 46.7%, VOL: 21.4%), and VL CSA (MD = 3.05 cm^2^; INT: 50%, VOL: 21.4%). Less than 10% of all participants experienced changes that exceeded the MD for MT (RF and VL) and CSA (RF, PM, and TB), while ∼31% of participants experienced changes that exceeded MD for %FAT (INT: 33.3%; VOL: 28.6%).

**Table 2:** Anthropometric changes and muscle hypertrophy following 8 weeks of training

		Covariate	POST	*F*	*P*-value	*η*^2^p	95% Confidence interval	
							Lower	Upper
Body mass (kg)	VOL	90.0	90.7 ± 13.2	0.802	0.379	0.030	89.4	92.0
	INT		91.2 ± 15.2				90.2	92.7
Body fat (%)	VOL	20.3	21.4 ± 6.0	0.040	0.843	0.002	19.6	21.0
	INT		19.7 ± 8.2				19.5	20.8
LBM (kg)	VOL	68.5	68.6 ± 7.9	0.570	0.457	0.021	68.3	70.5
	INT		69.9 ± 7.5				68.9	71.0
Lean arm mass (kg)	VOL	9.7	9.7 ± 1.1	4.816	0.037	0.156	9.6	10.1
	INT		10.3 ± 1.7				10.0	10.4
Lean leg mass (kg)	VOL	23.3	23.4 ± 3.0	0.226	0.638	0.009	23.2	24.2
	INT		23.8 ± 2.9				23.4	24.3
Rectus femoris								
MT (cm)	VOL	2.7	2.8 ± 0.3	0.212	0.649	0.008	2.6	2.8
	INT		2.6 ± 0.4				2.6	2.8
CSA (cm^2^)	VOL	16.0	16.8 ± 2.9	0.594	0.448	0.022	15.4	16.8
	INT		15.8 ± 4.9				15.8	17.1
Vastus lateralis								
MT (cm)	VOL	1.8	1.9 ± 0.3	0.578	0.454	0.022	1.8	2.0
	INT		1.9 ± 0.2				1.8	2.0
CSA (cm^2^)	VOL	38.8	40.1 ± 7.1	2.406	0.133	0.085	37.6	42.5
	INT		41.3 ± 9.6				40.2	45.0
Pectoralis major								
CSA (cm^2^)	VOL	78.9	77.1 ± 16.6	2.821	0.105	0.098	78.6	82.8
	INT		86.5 ± 13.7				81.1	85.1
Triceps brachii								
CSA (cm^2^)	VOL	10.1	11.7 ± 5.6	2.648	0.116	0.092	11.1	12.7
	INT		11.3 ± 5.6				10.3	11.8

CSA, cross-sectional area; LBM, lean body mass; MT, muscle thickness.

### Strength improvement

**Figure 1:**
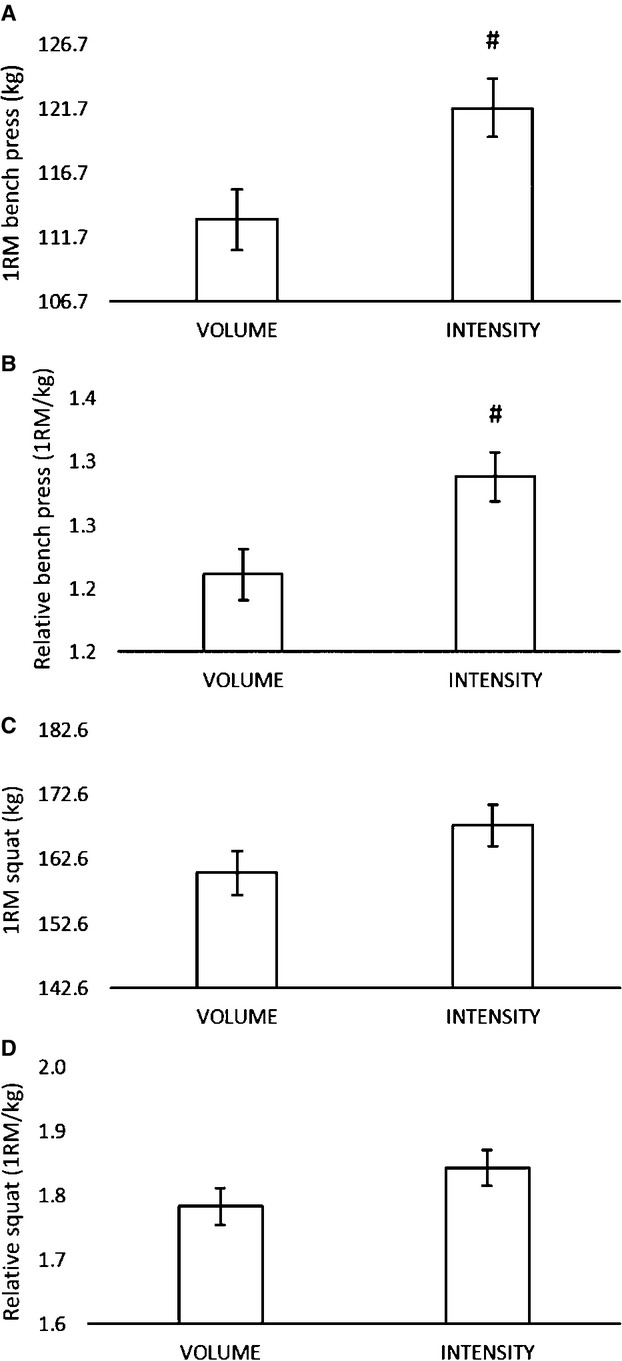
One repetition maximum (1RM) and relative bench press and squat strength. *Note*. Mean values (±SD) for posttest scores adjusted for initial differences in pretest: (A) bench press (covariate; adjusted pretest mean = 106.7); (B) relative bench press (covariate; adjusted pretest mean = 1.2); (C) squat (covariate; adjusted pretest mean = 142.6); (D) relative squat (covariate; adjusted pretest mean = 1.6). ^#^Significant (*P* < 0.05) difference between VOL and HVY.

Changes in bench press and squat 1RM are shown in Figure1. Significant improvements in 1RM bench press were observed in both VOL (PRE: 104.5 ± 19.2 kg, POST: 110.9 ± 17.5 kg, *P* = 0.018) and INT (PRE: 108.8 ± 31.8 kg, POST: 123.8 ± 34.1 kg, *P* < 0.001) groups, however the increase was significantly (*F* = 7.098, *P* = 0.013, *η*^2^ = 0.214) greater for INT than VOL (see Fig.1A). These findings were consistent when expressed relative to body mass (*F* = 7.558, *P* = 0.011, *η*^2^ = 0.225) (see Fig.1B). Collectively, significant (*P* < 0.001) improvements in absolute and relative 1RM squat were observed for both groups, with no differences occurring between groups (see Fig.1C and D).

### Electromyography

No group differences were observed for VL (VOL: 2.66 ± 1.18 V ˙ sec, INT: 3.70 ± 1.50 V ˙ sec, *P* = 0.053) or RF (VOL: 2.78 ± 1.93 V ˙ sec, INT: 3.47 ± 1.73 V ˙ sec, *P* = 0.333) activation at PRE, as determined from the slope of the regression (V ˙ sec ˙ %1RM^−1^) for muscle activation during increasing SQ intensity. Following the training intervention, the VL activation slope decreased for VOL (−0.43 ± 1.47 V ˙ sec ˙ %1RM^−1^) and INT (−1.62 ± 1.80 V ˙ sec ˙ %1RM^−1^), while RF activation also decreased for VOL (−1.09 ± 1.28 V ˙ sec ˙ %1RM^−1^) and INT (−1.72 ± 1.47 V ˙ sec ˙ %1RM^−1^). However, no group differences were observed (see Table3).

**Table 3:** Group differences in changes in muscle activation efficiency during submaximal and maximal squat assessment following 8 weeks of training

	Covariate	POST	*F*	*P*-value	*η*^2^p	95% Confidence interval	
						Lower	Upper
Vastus lateralis (*β*1)							
VOL	3.18	2.23 ± 1.78	0.664	0.423	0.026	1.603	3.160
INT		2.08 ± 0.84				1.152	2.708
Rectus femoris (*β*1)							
VOL	3.12	1.70 ± 1.17	0.468	0.500	0.018	1.396	2.251
INT		1.75 ± 0.83				1.193	2.048

Muscle activation efficiency was calculated as the percent change (*β*1) in muscle activation as resistance increased from 40% to 100% 1RM at PRE.

### Biochemical and hormonal responses

The biochemical response to resistance exercise (time course) during WK3 and WK10 are illustrated in Figure2, while the AUC responses at WK3 and WK10 are presented in Table4.

**Table 4:** Changes in the endocrine response (area under the curve) to exercise following 8 weeks of training

	Group means		Group × Time effect			Time effect	
	PRE	POST	*F*	*P*-value	*η*^2^p	t	*P*-value
Testosterone							
Volume	32.4 ± 6.5	32.7 ± 7.3	0.151	0.701	0.007	0.06	0.952
Intensity	36.4 ± 17.5	35.9 ± 20.2					
Cortisol							
Volume	2240.4 ± 716.4*	1769.8 ± 756.6#	7.604	0.011	0.241	2.703	0.019
Intensity	933.9 ± 555.5	1003.9 ± 557.8				−0.777	0.452
IGF-1							
Volume	318.7 ± 94.3	332.0 ± 96.6	6.020	0.021	0.194	−0.843	0.416
Intensity	407.6 ± 141.0	367.7 ± 133.4				2.676	0.019
Growth hormone							
Volume	23.6 ± 22.3*	9.1 ± 9.5	5.964	0.022	0.193	2.285	0.041
Intensity	3.6 ± 3.0	4.4 ± 3.8				0.595	0.562
Insulin							
Volume	300.1 ± 77.9	290.3 ± 70.0	0.379	0.544	0.015	1.198	0.242
Intensity	323.8 ± 131.6	291.8 ± 85.3					

* Significant (*P* < 0.05) group difference at PRE.

# Significant (*P* < 0.05) group difference at POST.

**Figure 2:**
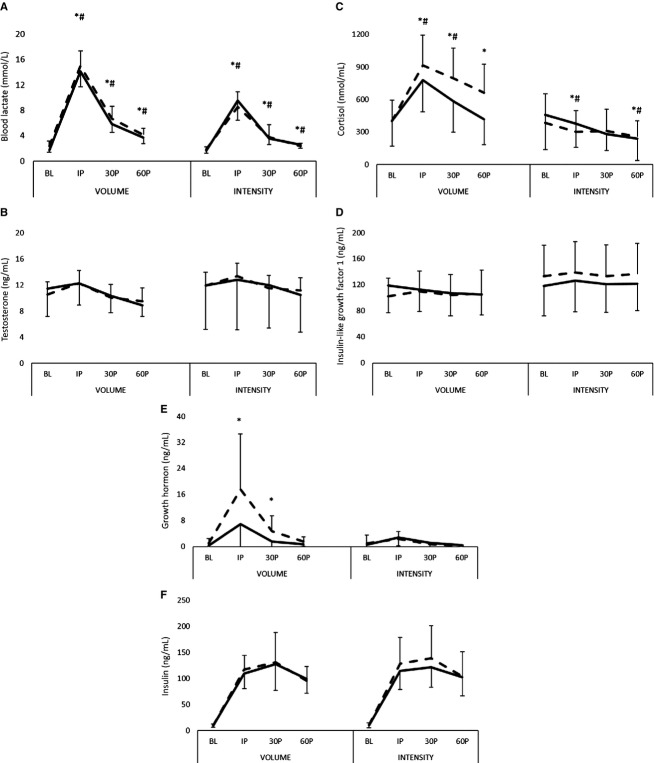
Changes in the biochemical response to exercise following 8 weeks of training. *Note*. (A) Lactate; (B) testosterone; (C) cortisol; (D) insulin-like growth factor-1; (E) growth hormone; (F) insulin. Pre- (PRE; dashed) and posttraining (POST; solid) values are presented as mean ± SD. *Significant (*P* < 0.05) difference from baseline at week 3. ^#^Significant (*P* < 0.05) difference from baseline at week 10. ^‡^Significant (*P* < 0.05) difference from week 3. ^†^Significant (*P* < 0.05) difference between VOL and INT.

### Lactate

Significant group × time interactions were observed in the lactate response to exercise at WK3 (*F* = 16.223, *P* < 0.001, *η*^2^ = 0.585) and WK10 (*F* = 12.679, *P* < 0.001, *η*^2^ = 0.524). Significant main effects (*P* < 0.001) were observed for both groups at WK3 and WK10. At WK3 blood lactate concentrations were significantly (*P* < 0.001) higher at IP compared to BL for both VOL (12.66 ± 2.31 mmol L^-1^) and INT (6.66 ± 2.44 mmol L^-1^) and remained significantly (*P* < 0.001) elevated from BL at 30P and 60P for both groups (see Fig.2A). Blood lactate concentrations were significantly (*P* < 0.001) higher for VOL than INT at each time point.

Elevations in blood lactate concentrations at WK10 for each group were similar to WK3. Blood lactate concentrations at IP in VOL (12.39 ± 2.22 mmol L^-1^) and INT (7.88 ± 3.17 mmol L^-1^) were significantly (*P* < 0.001) higher than BL. Blood lactate concentrations remained significantly (*P* < 0.001) elevated at 30P and 60P for both groups, but VOL experienced significantly (*P* < 0.002) greater elevations in blood lactate than INT at each time point.

### Testosterone

The TEST response to exercise for VOL and INT is shown in Figure2B. There were no group × time interactions observed at WK3 (*P* = 0.585) or WK10 (*P* = 0.286). However, significant main effects (*P* < 0.001) were observed when data were collapsed for both groups during WK3 and WK10. During WK3, TEST was significantly elevated from BL for both groups at IP (*P* < 0.001), but returned to resting levels by 30P and dropped significantly (*P* < 0.001) below BL at 60P. During WK10, TEST remained similar to BL at IP and 30P, but decreased significantly (*P* = 0.002) below BL at 60P. AUC analysis (Table4) did not reveal a significant group × time interaction or time effect from training.

### Cortisol

The CORT responses to exercise are shown in Figure2C. A significant group × time interaction was observed at WK3 (*F* = 8.687, *P* = 0.002, *η*^2^ = 0.441) and WK10 (*F* = 5.922, *P* = 0.009, *η*^2^ = 0.350). At WK3, CORT concentrations for VOL were significantly elevated (*P* < 0.001) from BL at IP, 30P, and 60P. In contrast, CORT concentrations in INT were significantly reduced at IP (*P* = 0.026), but returned to BL at 30P (*P* = 0.089), and then further declined at 60P (*P* = 0.032). At WK10, CORT concentrations in VOL were significantly elevated from BL at IP (*P* < 0.001) and at 30P (*P* = 0.020), but returned to BL by 60P (*P* = 0.768). In INT, CORT concentrations did not differ at IP (*P* = 0.278), but were significantly reduced at 30P (*P* = 0.023) and 60P (*P* = 0.001).

AUC analysis (Table4) for the CORT response revealed a significant group × time interaction. At WK3, the acute CORT response for VOL was significantly greater (*P* < 0.001) than INT. In comparison to WK3, VOL experienced a significantly (−13.1 ± 17.8%) attenuated response during WK10, while the response for INT remained similar. However, the CORT response for VOL was still greater (*P* = 0.007) than INT at WK10.

### Insulin-like growth factor 1

The IGF-1 responses to exercise are depicted in Figure2D. There were no group × time interactions observed at WK3 (*P* = 0.966) or WK10 (*P* = 0.899). When data from both groups were collapsed, a significant main effect was observed at WK3 (*P* = 0.003), but not at WK10 (*P* = 0.185). During WK3, IGF-1 was significantly elevated from BL at IP (*P* < 0.001), but returned to BL by 30P. AUC analysis (Table4) for the acute IGF-1 response to exercise revealed a significant group × time interaction. Although no differences (*P* = 0.068) were observed between the AUC for VOL and INT at WK3, a significant attenuated response (−9.4 ± 12.6%) was seen for INT at WK10. However, no changes were noted in the AUC response from WK3 to WK10 for VOL. No differences (*P* = 0.436) were observed between VOL and INT at WK10.

### Growth hormone

The GH responses to exercise are shown in Figure2E. During WK3, significant group × time interactions (*F* = 5.411, *P* = 0.012, *η*^2^ = 0.320) were observed. GH concentrations for VOL were significantly elevated from BL at IP (*P* = 0.003) and 30P (*P* = 0.013), then returned to BL by 60P (*P* = 0.245). In contrast, no changes in GH concentrations were observed in INT. During WK10, no group differences (*F* = 3.262, *P* = 0.057, *η*^2^ = 0.221) were observed in the GH response. When data were collapsed, a significant main effect (*P* = 0.005) was observed. GH concentrations for both groups were elevated from BL at IP (*P* < 0.001) and 30P (*P* = 0.011), but returned to baseline by 60P.

AUC analysis (Table4) for the GH response to exercise revealed a significant group × time interaction. At WK3, the GH response for VOL was significantly greater (*P* = 0.007) than INT. In comparison to WK3, VOL experienced a significantly (−55.7 ± 29.7%) diminished response during WK10, while the response for INT remained the same. The GH responses for VOL and INT at WK10 were not statistically (*P* = 0.119) different.

### Insulin

The INSL responses to exercise are shown in Figure2F. There were no significant group × time interactions observed at WK3 (*P* = 0.960) or WK10 (*P* = 0.823). However, significant main effects (*P* < 0.001) were observed at WK3 and WK10 when collapsed across groups. The INSL responses for both VOL and INT were significantly (*P* < 0.001) elevated from BL at every time point during both WK3 and WK10 assessments. No significant interactions were noted between VOL and INT in WK3 and WK10 comparisons. AUC analysis (Table4) did not reveal a significant group × time interaction or time effect from training on the INSL response to exercise.

### Nutritional intake and dietary analysis

Relative kilocaloric intake did not change significantly over the course of the investigation for VOL (PRE: 31.7 ± 7.0 kCal ˙ kg^−1^; POST: 29.2 ± 8.1 kCal ˙ kg^−1^) or INT (PRE: 38.3 ± 11.1 kCal ˙ kg^−1^; POST: 31.1 ± 5.3 kCal ˙ kg^−1^). Similarly, relative protein intake also remained consistent for VOL (PRE: 1.8 ± 0.4 g ˙ kg^−1^; POST: 1.7 ± 0.7 g ˙ kg^−1^) and INT (PRE: 2.0 ± 0.7 g ˙ kg^−1^; POST: 1.7 ± 0.4 g ˙ kg^−1^). In addition, no differences were observed between groups at PRE or POST.

## Discussion

The major findings of this study indicated that 8 weeks of high-intensity, low-volume resistance training utilizing long rest intervals stimulated significantly greater 1RM bench press and lean arm mass gains compared to moderate intensity, high-volume program utilizing short rest intervals in resistance-trained men. These results are consistent with previous comparative studies in resistance-trained individuals showing high-intensity programs were more conducive for increasing strength while producing similar magnitude of muscle hypertrophy (Brandenburg and Docherty 2002; Schoenfeld et al. 2014). However, the greater gains in some measures of muscle size observed in INT indicate that high-intensity training may provide a greater stimulus for muscle hypertrophy in trained men. In addition, the hormonal responses for testosterone were similar between groups. At WK3, the GH and cortisol responses were greater for VOL compared to INT. Although both the GH and cortisol responses in VOL were attenuated after 8 weeks of training, the cortisol response remained greater. No differences were noted in the IGF-1 and insulin responses between groups in addition to the testosterone response.

Changes in strength are generally thought to be the result of a combination of neurological activation and skeletal muscle adaptation (Moritani and deVries 1979; Moritani 1993). Initial strength gains in previously untrained individuals have been associated with neurological adaptations that involve a more efficient activation pattern of activated musculature (Moritani and deVries 1979). However, improvements in the magnitude and efficiency of muscle activation during exercise appear to be limited in resistance-trained populations (deVries 1968; Moritani and deVries 1979; Moritani 1993). Consequently, any change in muscle activation is likely the result of a change in muscle size. A previous study in resistance-trained athletes reported significant strength gains within the first few weeks of training (Hoffman et al. 2009). This was attributed to the participants entering the intervention following several weeks of detraining and rapidly adapting to the familiar training stimulus. To minimize any “relearning effect,” a 2-week “pretraining” period was used prior to any performance assessments in the present study. As such, any change in muscle activation is likely the result of the specific training program and not related to a relearning effect.

Results of this study noted similar changes in muscle activation efficiency for both groups, as well as similar changes in strength and size of the lower extremity. These findings are in agreement with a previous study examining improvements in muscle activation efficiency following 12 weeks of isometric training (Komi et al. 1978). Although no differences were observed between groups, it is possible that 8 weeks was not sufficient to reveal differences in the lower extremity of resistance-trained men (Abe et al. 2000; Chen et al. 2011). Unlike the upper extremity, where group differences in strength gains and hypertrophy were observed, the musculature of the lower limbs have been observed to be more resistant to exercise-induced muscle damage (Chen et al. 2011) and slower to respond to training (Abe et al. 2000). Thus, a longer training period may be necessary to determine whether high-intensity or high-volume training is more advantageous for inducing lower extremity strength and size improvements in an experienced population.

The mechanical and metabolic stresses imposed by resistance training are believed to influence changes in muscle size (deVries 1968; Moritani and deVries 1979; Moritani 1993). In this study, the greater mechanical stress imposed by INT was also accompanied by greater gains in upper body muscle growth. This contrasts with previous investigations which showed that the mechanical and metabolic stresses of high-volume and high-intensity training programs provided a similar stimulus for muscle growth in trained participants (Brandenburg and Docherty 2002; Schoenfeld et al. 2014). These differences may be explained, in part, by the level of tissue activation associated with the exercise selection (Tipton et al. 2001; Goto et al. 2004). Notably, the limited number of multiple joint structural exercises used per training session in those studies. Brandenburg and Docherty (2002) used only two single-joint, open-chain exercises (i.e., preacher curl and supine elbow extension) per session, while Schoenfeld et al. (2014) required three exercises per session (i.e., 1 × upper body push, 1 × upper body pull, and 1 × lower body movement) out of a pool of nine exercises that included both single- and multiple joint movements. Although speculative, it is possible that the greater morphological changes observed in INT were the consequence of greater muscle activation generated from the inclusion of several multiple joint exercises per workout. The higher intensity protocol likely activated more muscle fibers during exercise (Henneman et al. 1965), stimulating greater adaptation across a larger percentage of muscle (Clarkson et al. 1992; Ratamess et al. 2009).

The acute endocrine responses observed in our study were consistent with previous investigations (Kraemer et al. 1990; Hakkinen and Pakarinen 1993; Schwab et al. 1993; Hakkinen et al. 2000; Hameed et al. 2004; McKay et al. 2008; McCaulley et al. 2009; West et al. 2009, 2010; Gregory et al. 2013). The high-volume resistance training protocol resulted in significantly greater elevations in GH and cortisol concentrations, compared to the high-intensity training protocols, while similar increases were observed between the training protocols in the testosterone, IGF-1, and insulin responses to exercise. Elevations in the anabolic hormones testosterone, GH, and IGF-1 are thought to be advantageous for muscle growth and strength gain (Kraemer and Ratamess 2005; Ratamess et al. 2009). The elevations in GH and cortisol observed in VOL were likely in response to the metabolic demands of the exercise protocol, as reflected by the significantly higher lactate measures seen in VOL compared to INT (Moller et al. 1995; Schakman et al. 2013). Furthermore, the effect of 8 weeks of training appeared to attenuate both the GH and cortisol responses to exercise in VOL. While this is in contrast to previous studies (McCall et al. 1999; Hakkinen et al. 2000; Buresh et al. 2009; Mitchell et al. 2013), it may reflect metabolic adaptations to the exercise stimulus (Burgomaster et al. 2003; Kraemer and Ratamess 2005). The elevated GH observed at IP (WK10) for INT may reflect the increase in absolute load being used, making this program metabolically more stressful; though it was not sufficient to alter the cortisol response.

The similar responses of testosterone and IGF-1 observed between the training protocols, at both WK3 and WK10, suggest that differences in acute program variables (i.e., intensity, volume, and rest) may not stimulate significant differences in the response of these anabolic hormones when assessed only the first and last week of an 8-week training program. Previous investigations have reported a similar testosterone response following both heavy (3–6 RM) and moderate (9–10 RM) loading schemes (Kraemer et al. 1990; Schwab et al. 1993; McCaulley et al. 2009), while a consistent response pattern has not been observed for IGF-1 in response to a variety of high-volume resistance exercise protocols (Nindl et al. 2001; Wilkinson et al. 2006; McKay et al. 2008; Spiering et al. 2008; West et al. 2009, 2010; Gregory et al. 2013). Variability in the testosterone response is likely related to the degree of mechanical stress present (i.e., loading). Kraemer et al. (1990) have previously demonstrated elevated testosterone concentrations only when heavy (i.e., 5RM with 1- or 3-min rest) or moderate (i.e., 10RM with 1-min rest) loadings were used to induce fatigue. When moderate loads and long rest intervals (i.e., 10RM with 3-min rest) were used, this stimulus did not appear to be sufficient to cause consistent elevations in testosterone concentrations. Similarly, IGF-1 concentrations have been reported to remain at baseline concentrations following high-volume resistance exercise when the exercise protocol consisted of only two unilateral exercises with a long rest period (6–10 RM; 3-min rest) (Wilkinson et al. 2006), or when training included both high (5RM) and moderate (10RM) intensity with longer rest periods (2–3 min) (Nindl et al. 2001; Spiering et al. 2008). The variability seen in the acute testosterone and IGF-1 response to a bout of resistance exercise suggests that different combinations of both metabolic and mechanical stimuli may be required to foster such changes.

Elevations in IGF-1 concentrations are thought to be stimulated by a high mechanical stimulus (Bamman et al. 2001; Hameed et al. 2004; Gregory et al. 2013). However, elevations in GH have also been reported to stimulate IGF-1 release (Hameed et al. 2004; Iida et al. 2004; Gregory et al. 2013). In this study, IGF-1 concentrations were similarly elevated between groups at WK3 and WK10 though GH concentrations were only similar between groups at WK10. In VOL, an attenuated GH (AUC) response was noted following training, while the IGF-1 (AUC) response remained consistent. In contrast, the response seen for INT was slightly different; the GH (AUC) response remained consistent, while IGF-1 (AUC) was attenuated. Nevertheless, both observations question the role that GH has on the IGF-1 response to acute exercise. Furthermore, these results are consistent with other investigations that have reported dissimilar responses from GH to IGF-1 following resistance exercise (Wilkinson et al. 2006; Spiering et al. 2008; Hasani-Ranjbar et al. 2012). Interestingly, Hameed et al. (2004) reported that elevations in GH (exogenous administration) combined with a resistance exercise stimulus resulted in significant elevations in both circulating and intramuscular IGF-1. Although the combination of both GH administration and resistance exercise resulted in a greater IGF-1 response than any of the stimuli alone, 12 weeks of GH administration was reported to increase intramuscular IGF-1 concentrations and attenuate circulating IGF-1 concentrations. Although intramuscular IGF-1 was not examined in this study, this may provide some explanation for the attenuation in the IGF-1 response to INT at WK10.

Unlike the GH and IGF-1 responses to exercise, which appear to be influenced by changes in metabolic stress (Vanhelder et al. 1984; Hakkinen and Pakarinen 1993) and possibly changes in GH concentration (Hameed et al. 2004; Iida et al. 2004; Gregory et al. 2013), respectively, the mechanisms underlying the changes in the testosterone response to exercise are less clear. Previous research has reported no changes in the testosterone response to exercise (McCall et al. 1999; Bell et al. 2000; Hakkinen et al. 2000; Wilkinson et al. 2006) or an attenuated response following prolonged (2–6 months) training (Buresh et al. 2009; West et al. 2010; Mitchell et al. 2013). In the present study, neither protocol induced any changes in the testosterone response to exercise. These results are consistent with previous research using high-volume (McCall et al. 1999; Bell et al. 2000; Wilkinson et al. 2006) and high-intensity (Bell et al. 2000) resistance training protocols for 8–12 weeks in duration. While it may be possible that the 8-week training period used in this study was too short to stimulate any adaptation, the testosterone response to an acute bout of resistance exercise has also been reported to remain similar for up to 6 months of training (Hakkinen et al. 2000). Others have reported a reduced response following 10–16 weeks of high-volume training (Buresh et al. 2009; West et al. 2010). Consequently, there does not appear to be a clear pattern or mechanism of change in the testosterone response to resistance training.

Insulin concentrations in both groups were shown to be significantly elevated from BL at IP through 60P during both WK3 and WK10. While this response is in contrast to many studies showing insulin concentrations decreasing from baseline during exercise (Thyfault et al. 2004; Spiering et al. 2008), these differences may be related to the feedings provided during the study. All participants were provided ∼235 mL of chocolate milk (or Lactaid ®) following baseline blood sample collection (before exercise), and immediately following the IP blood draw. Previous research has demonstrated that ingestion of a protein/carbohydrate beverage surrounding the workout will result in an elevation in insulin concentrations (Tipton et al. 2001; Thyfault et al. 2004). It is possible that any differences in the insulin response to the different training protocols may have been masked by the pre- and postexercise feedings.

In conclusion, the results of this study indicate that high-intensity (3–5 RM), low-volume resistance training program utilizing a long rest interval (3 min) is more advantageous than a moderate intensity, high-volume (10–12 RM) program utilizing a short rest interval (1 min) for stimulating upper body strength gains and muscle hypertrophy in resistance-trained men during an 8-week study. Furthermore, the strength and morphological improvements demonstrated did not appear to be influenced by the endocrine response. These observations question the utility of high-volume training programs that are designed to maximize the acute hormonal response as being ideal for stimulating muscle growth, at least during a relatively short duration of training. Emphasizing training intensity over volume may provide an advantage for accelerating muscle growth and strength gains in a short-term training cycle.
